# Characterization of Chlorophyll Fluorescence and Antioxidant Defense Parameters of Two *Gracilariopsis lemaneiformis* Strains under Different Temperatures

**DOI:** 10.3390/plants12081670

**Published:** 2023-04-17

**Authors:** Xiaomei Li, Xue Meng, Xiaoqi Yang, Delin Duan

**Affiliations:** 1 CAS and Shandong Province Key Laboratory of Experimental Marine Biology, Center for Ocean Mega-Science, Institute of Oceanology, Chinese Academy of Sciences, Qingdao 266071, China; 2 Laboratory for Marine Biology and Biotechnology, Pilot National Laboratory for Marine Science and Technology (Qingdao), Qingdao 266071, China; 3 University of Chinese Academy of Sciences, Beijing 100049, China

**Keywords:** antioxidant enzymes, chlorophyll fluorescence, Rhodophyta

## Abstract

In this study, two *Gracilariopsis lemaneiformis* strains—the wild type and a green-pigmented mutant—were cultured at three temperatures (8, 20, and 30 °C) for 7 days to explore their temperature tolerance using photosynthetic performance and antioxidant defense parameters. When the two strains of *G. lemaneiformis* were separately cultured at 30 °C, the fast chlorophyll fluorescence intensity of the wild type decreased, whereas the green mutant showed no significant change. The decrease in the performance index on absorption basis value under heat stress was lower in the green mutant than in the wild type. In addition, the green mutant had stronger antioxidant activity at 30 °C. Furthermore, a greater decrease in the values of maximum photochemical quantum yield and performance index on an absorption basis in the green mutant indicated that it had a greater degree of inhibition of photosynthetic performance under low temperatures. However, the green mutant produced less reactive oxygen species under low temperatures, suggesting that the antioxidant potential of the green mutant might be higher. In conclusion, the green mutant exhibited heat tolerance and could recover from low-temperature damage; therefore, it has the potential for large-scale cultivation.

## 1. Introduction

*Gracilariopsis lemaneiformis*, an economically important seaweed, has been widely cultivated in China for over 20 years [1]. According to the National Bureau of Statistics [2], the annual yield of Gracilariales (mainly *G. lemaneiformis*) is the second largest in China. Besides being the main source of agar extraction [3], the alga has been widely used as fodder for abalone aquaculture and is ideal for carbon assimilation to remediate eutrophic ecosystems [4,5,6]. In addition, polysaccharides and their derivatives obtained from *G. lemaneiformis* have been found to perform various biological activities such as prebiotic, antiradiation, antitumor, immunomodulatory, and antioxidant activities [7,8,9].

Temperature is one of the key factors affecting the physiology and distribution of seaweeds [10]. Temperature can affect agar yield and biochemical components of *G. lemaneiformis* [11]. The suitable temperature for the growth of the wild population of *G. lemaneiformis* was between 12 °C and 23 °C [12]. Temperatures above 23 °C can disrupt the metabolic processes of *G. lemaneiformis* or even hinder its normal growth and development, leading to disease, death, and yield reduction [13,14]. The industrial production of *G. lemaneiformis* is limited by high temperatures in summer, especially along the coast of the South China Sea [15]. The heat-tolerant cultivars 981 and 2007, which can tolerate temperatures ranging from 23 °C to 26 °C, are widely cultivated in the South China Sea coastal region [3,16]. However, these cultivars still cannot survive in summer. With the increase in global warming, the temperature tolerance of *G. lemaneiformis* needs to be explored to improve its germplasm for aquaculture. Besides temperature, other factors, such as light and nutrient supply, are important for the growth of *G. lemaneiformis* [17].

Photosynthesis is one of the most sensitive physiological responses of plants, including seaweeds, to environmental changes. In the photosynthetic electron-transfer reaction, light energy fuels the electron transport from water to NADP+ via photosystem II (PSII), cytochrome b6f (Cyt b6f) complex, and photosystem I (PSI) to generate NADPH [18]. PSII is a multisubunit pigment transmembrane protein complex on the thylakoid membrane, consisting of D1, D2, CP43, and CP47 proteins [19]. PSII is sensitive to a wide range of environmental conditions and susceptible to damage; especially, the D1 protein is vulnerable to attack by reactive oxygen species (ROS) generated by reaction centers (RCs) [20]. Chlorophyll fluorescence is a non-invasive, reliable, and effective technique to assess the changes in photosynthetic performance, especially under stress conditions [21,22]. Moreover, chlorophyll fluorescence parameters are widely used to assess photosynthetic features in algae [23,24,25]. They have been used to study the stresses of temperature, carbon dioxide, light, and nutrient influence in *G. lemaneiformis* and to estimate the degree of stress on its photosynthetic apparatus under environmental conditions [26]. Using polyphasic chlorophyll fluorescence transients (OJIP) analysis, it was found that *G. lemaneiformis* was a more temperature-dependent than nutrient-dependent species [27]. The significant decrease in *F*_V_/*F*_m_ of *G. lemaneiformis* at an irradiance of 20 μmol photons m^−2^ s^−1^ (low light) indicated a decrease in its photosynthetic efficiency, which to some extent explained the mechanism of light irradiance stress inhibiting the growth of *G. lemaneiformis* [28]. Therefore, chlorophyll fluorescence parameters form a sensitive index for reflecting marine environmental influences on seaweed physiological processes.

It is known from various marine organisms that environmental perturbations, such as cold, heat, and oxidative stress, lead to the formation of ROS [29]. A moderate amount of ROS acts as a signal to induce an increase in antioxidant capacity, thereby increasing cellular stress resistance and viability [30]. However, excess amounts of ROS in seaweeds can cause damage to DNA, proteins, and membrane lipids, especially to the photosynthetic apparatus [31,32,33]. ROS was rapidly generated when *G. lemaneiformis* was exposed to heat stress [15]. Enzymatic antioxidant defenses, such as superoxide dismutase (SOD), peroxidase (POD), and catalase (CAT), can scavenge ROS in seaweeds [6,28].

In the large-scale cultivation of *G. lemaneiformis*, excellent cultivar and germplasm are important factors to ensure its quality and yield, and the selection and promotion of *G. lemaneiformis* is a prerequisite for large-scale farming [34]. The acquisition of mutants helps in the search for superior germplasm. The *G. lemaneiformis* cultivar 981 was an excellent strain obtained from culture trials after nearly 20 years of domestication and screening of wild populations of *G. lemaneiformis*, which had the characteristics of high-temperature tolerance and high growth rate. The *G. lemaneiformis* cultivar 07-2 was obtained from the cultivar 981 by using N-methyl-N′-nitro-N-ni-trosoguanidine (MNNG), which has excellent characteristics of rapid growth and greater resistance to harsh environments [35]. The *G. lemaneiformis* cultivar Lulong No.1 is an excellent cultivar obtained by population selection, single cross-breeding, and UV mutagenic high-temperature stress screening using wild populations of *G. lemaneiformis* as the parent [36]. Huang et al. [37] analyzed the gene related to optically active phycoerythrin synthesis in both wild-type and green-pigmented mutant, and they found a reduction in optically active phycoerythrin in the green mutant. However, stress tolerance analyses of the *G. lemaneiformis* wild type and mutant have not been reported.

In the present study, the physiological responses of a green-pigmented mutant and the wild type of *G. lemaneiformis* to heat and cold stresses were analyzed using chlorophyll fluorescence and antioxidant defense parameters. The objectives of this study were (1) to compare the temperature tolerance between the wild type and the green mutant and (2) to provide a research base for the selection of high-quality *G. lemaneiformis* germplasm resources.

## 2. Results

### 2.1. Molecular Phylogenetic Analysis

**Figure 1:**
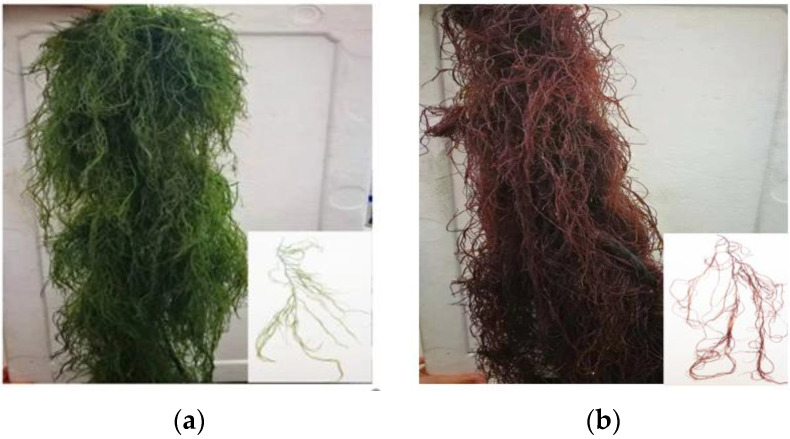
Two *G. lemaneiformis* strains. Green-pigmented mutant (**a**) and wild type (**b**).

**Figure 2:**
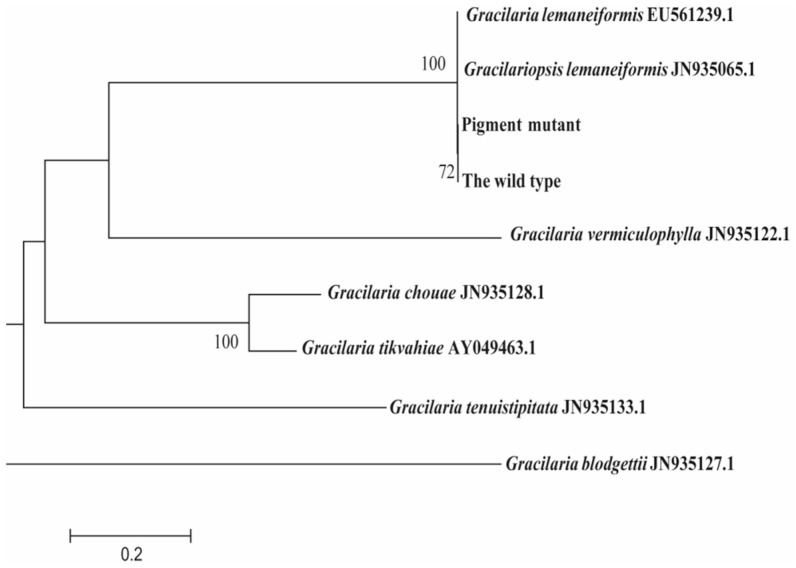
Molecular phylogenetic analysis by maximum likelihood method based on internal transcribed spacer (ITS).

The stable green-pigmented mutant *(*Figure 1a) was derived from the *G. lemaneiformis* cultivar 981 (Figure 1b) by spontaneous mutation and continuous selection for 3 years from 2019. Moreover, the molecular phylogenetic analysis (Figure 2) indicated that the green mutant and the wild type grouped with referenced *G. lemaneiformis*, forming a unique clade.

### 2.2. OJIP Fluorescence Transient Analysis

**Figure 3:**
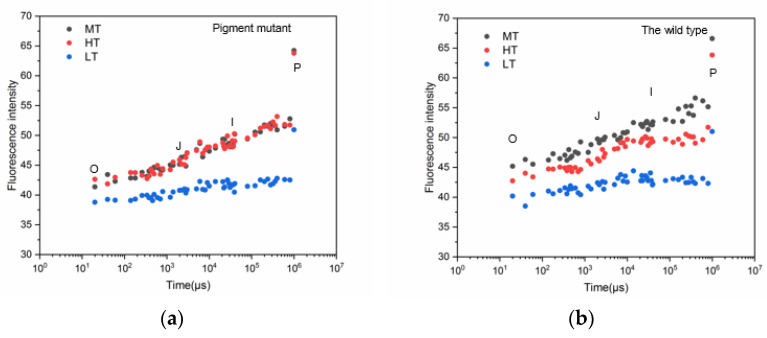
OJIP transient curves in the green mutant (**a**) and wild type (**b**) of *G. lemaneiformis* exposed to three temperature levels for 7 days. LT, low temperature 8 °C (blue). MT, medium temperature 20 °C (black). HT, high temperature 30 °C (red). MT was the control.

The OJIP transient curves of the two strains exhibited a polyphasic rise of O (20 μs), J (2 ms), I (30 ms), and P (maximum fluorescence) (Figure 3). After heat stress, the OJIP transient of the green mutant was similar to that at room temperature, indicating a better adaptation of the green mutant to heat stress (Figure 3a). However, all steps of the OJIP transient curve of the wild type showed a downward trend for heat stress conditions compared with normal temperature conditions (Figure 3b). In both strains, all steps of the OJIP transient curves were much lower for low-temperature conditions than for normal-temperature conditions. The lower fluorescence intensity of the OJIP transient in the two strains under low temperatures indicated an inhibition of photosynthetic performance.

### 2.3. Temperature Influences on Photosynthetic Electron Transport

**Figure 4:**
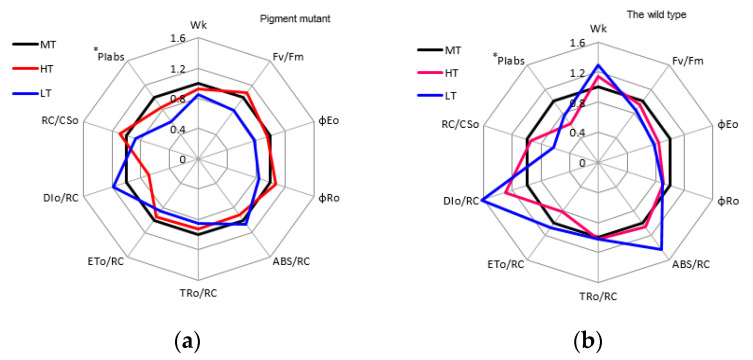
Spider plots of JIP-test parameters deduced from chlorophyll a fluorescence OJIP transient curves in the green mutant (**a**) and wild type (**b**) of *G. lemaneiformis* exposed to three temperature levels for 7 days. LT, low temperature 8 °C (blue). MT, medium temperature 20 °C (black). HT, high temperature 30 °C (red). The differences between treatments (HT and LT) and the control (MT) were considered significant at * *p* < 0.05.

In the green mutant, damage to the oxygen-evolving complex (*W*_K_), quantum yield for electron transport (*φ*_Eo_), ABS/RC, TRo/RC, ETo/RC, DIo/RC, and performance index (PI_abs_) values exhibited a downward trend, whereas maximum photochemical quantum yield (*F*_V_/*F*_m_), the quantum yield of reduction of end electron acceptors of PSI (*φ*_Ro_), and RC/CSo values exhibited an upward trend after exposure to heat stress compared with normal temperature conditions (Figure 4a, Table S1).

In the wild type of *G. lemaneiformis*, *F*_V_/*F*_m_, *φ*_Eo_, *φ*_Ro_, ETo/RC, RC/CSo, and PI_abs_ values exhibited a downward trend, whereas *W*_K_, ABS/RC, and DIo/RC values exhibited an upward trend under heat stress compared with normal temperature conditions (Figure 4b). The increase in *W*_K_ in the wild type indicated that the oxygen-evolving complex (OEC) might be damaged.

PI_abs_ values of the two strains decreased after being exposed to heat stress (*p* < 0.05). Moreover, the PI_abs_ value of the green mutant dropped by 16%, and that of the wild type decreased by 37% (*p* < 0.05), demonstrating that the PSII overall performance was less affected by heat stress in the green mutant than in the wild type. Therefore, high-temperature tolerance may be higher in the green mutant than in the wild type.

In the green mutant, *W*_K_, *F*_V_/*F*_m_, *φ*_Eo_, *φ*_Ro_, TRo/RC, ETo/RC, RC/CSo, and PI_abs_ values exhibited a downward trend, whereas ABS/RC and DIo/RC values exhibited an upward trend after exposure to low temperature compared with normal temperature conditions. In the wild type, *F*_V_/*F*_m_, *φ*_Eo_, *φ*_Ro_, RC/CSo, and PI_abs_ values exhibited a downward trend, whereas *W*_K_, ABS/RC, TRo/RC, ETo/RC, and DIo/RC values exhibited an upward trend under low temperature compared with normal-temperature conditions. Under low temperature, the *F*_V_/*F*_m_ value of the green mutant decreased by 22%, and that of the wild type decreased by 14%. The PI_abs_ value decreased by 40% in the green mutant and 24% in the wild type (*p* < 0.05). The greater decrease in the *F*_V_/*F*_m_ and PI_abs_ values in the green mutant indicated that it had a greater degree of inhibition of photosynthetic performance.

### 2.4. Non-Photochemical Quenching Detection

**Figure 5:**
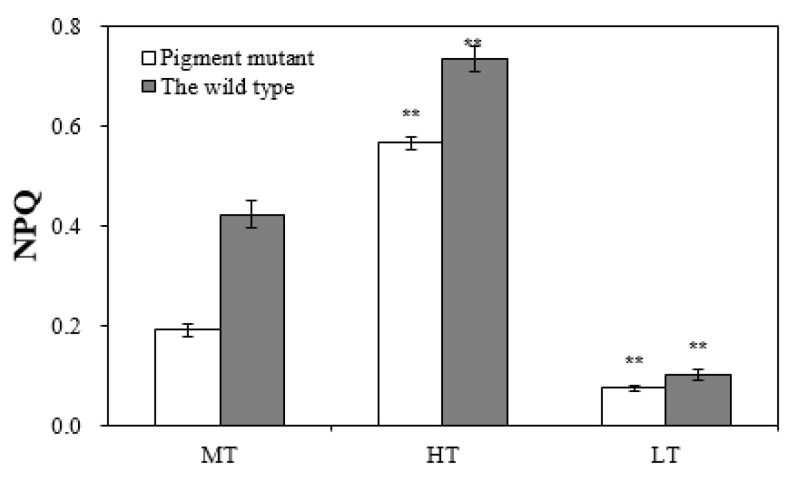
Non-photochemical quenching in the green mutant and wild type of *G. lemaneiformis* exposed to three temperature levels for 7 days. LT, low temperature 8 °C. MT, medium temperature 20 °C. HT, high temperature 30 °C. The differences between treatments (HT and LT) and the control (MT) were considered significant at ** *p* < 0.01.

With exposure to heat stress, the non-photochemical quenching (NPQ) value increased three-fold (from 0.19 to 0.57) in the green mutant and nearly two-fold (from 0.42 to 0.73) in the wild type (Figure 5). The lower increase in the NPQ value in the wild type indicated a greater suppression of the photo-protection of NPQ. Interestingly, the NPQ value of the wild type under heat stress was 1.3-fold that of the green mutant. In addition, NPQ values in both strains were lower under low temperatures (Figure 5).

### 2.5. Oxygen-Free Radical and Lipid Peroxidation Measurements

**Figure 6:**
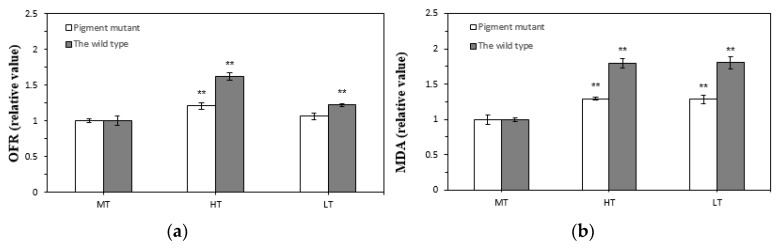
Superoxide content (OFR, (**a**)) and lipid peroxidation level (MDA, (**b**)) of *G. lemaneiformis* exposed to three temperature levels for 7 days. LT, low temperature 8 °C. MT, medium temperature 20 °C (control). HT, high temperature 30 °C. The differences between treatments (HT and LT) and the control (MT) were considered significant at ** *p* < 0.01.

OFR and MDA (lipid peroxidation level) content of the two strains were significantly high after heat stress compared with normal temperature conditions (Figure 6, *p* < 0.01). The OFR content in the green mutant and wild type increased by 21% and 62%, respectively, indicating that the wild type produced more ROS under heat stress. Moreover, the MDA content in the green mutant and wild type increased by 29% and 80%, respectively, showing that the green mutant had less lipid peroxidation, whereas the wild type had severe lipid peroxidation under heat stress.

The OFR content did not significantly change in the green mutant, but it significantly increased in the wild type after low-temperature treatment (Figure 6a, *p* < 0.01). The MDA content increased by 28% in the green mutant and 80% in the wild type (Figure 6b, *p* < 0.01), indicating more lipid peroxidation in the wild type under low temperature.

### 2.6. Antioxidant Enzyme Activity Measurements

**Figure 7:**
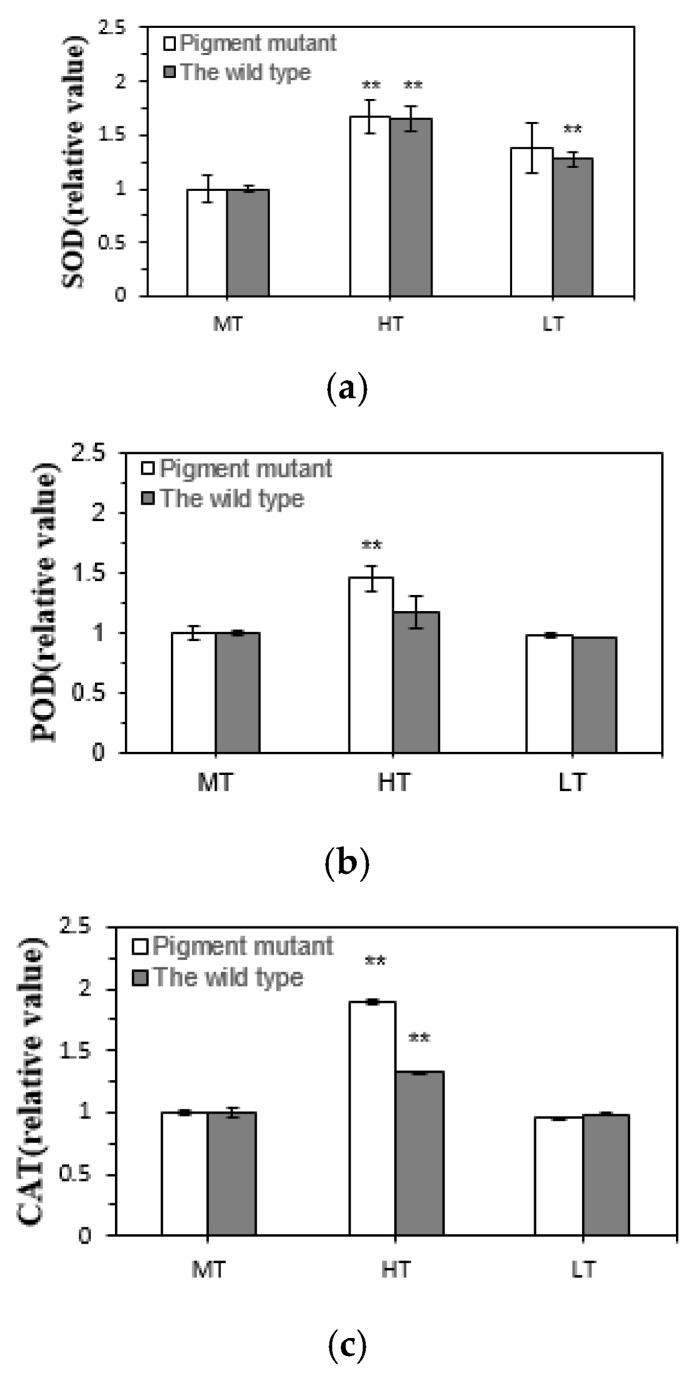
Superoxide dismutase (SOD, (**a**)), peroxidase (POD, (**b**)), and catalase (CAT, (**c**)) activities in the two strains of *G. lemaneiformis* exposed to three temperature levels for 7 days. LT, low temperature 8 °C. MT, medium temperature 20 °C (control). HT, high temperature 30 °C. The differences between treatments (HT and LT) and the control (MT) were considered significant at ** *p* < 0.01.

The SOD, POD, and CAT activities of the two *G. lemaneiformis* strains increased under heat stress (Figure 7). The increased SOD (*p* < 0.01), POD (*p* < 0.01), and CAT (*p* < 0.01) activities in the green mutant indicate that it had a more efficient antioxidant system to protect from oxidative stress compared with the wild type. The low temperature did not significantly change POD and CAT activities in the two strains. There was a non-significant upward trend in SOD activity (*p* > 0.05) in the green mutant after low-temperature stress, while there was a weak increase in SOD activity (*p* < 0.01) in the wild type.

## 3. Discussion

### 3.1. Heat Stress in G. lemaneiformis

High summer temperatures can lead to algal decay, which limits the large-scale cultivation of *G. lemaneiformis* in China [38]. The two *G. lemaneiformis* strains showed different responses to heat stress. In plants and algae, PSII is the most sensitive component of the electron transport chain of photosynthesis in response to environmental changes [39]. OJIP fluorescence transient reflects the changes in the primary photochemical reaction of PSII and the electron transfer state of the photosynthetic structure [22]. The fluorescence intensity of the OJIP transient decreased in the *G. lemaneiformis* wild type at 30 °C, showing that high temperature may inhibit electron transfer on the PS II donor side or the PS II acceptor side. However, no significant effect was observed in the green mutant, indicating that the green mutant showed acclimation of photosynthesis under heat stress. To further investigate the acclimation of photosynthesis under heat stress in the green mutant, we calculated several JIP parameters that identify sensitive functions such as energy absorption, energy capture, and electron transport in the PS II and PS I [40].

The *F*_V_/*F*_m_ values of the two strains showed no significant difference as the temperature increased from 20 °C to 30 °C. However, heat stress resulted in an apparent decrease in the PI_abs_ value in both wild type and mutant. These results suggested that PI_abs_ was more sensitive to stress compared to *F*_V_/*F*_m_, which was consistent with previous reports [41]. PI_abs_ is a multiparametric expression and was highly used to identify plant vitality exposed to environmental stress, and it takes into consideration the three main functional steps: the density of active reaction centers per chlorophyll; the ratio of the de-excitation rate constants for photochemical and non-photochemical events; and the efficiency of the conversion of excitation energy to electron transport toward the plastoquinone pool [21,42]. A large decrease in PI_abs_ of the wild type indicated the destructive effects of heat stress on PSII. While the small decrease in the PI_abs_ value in the green mutant indicated that the PSII overall performance was less affected by heat stress in the green mutant than in the wild type.

Heat stress can block PSII reaction centers (RCs) and dissociate antenna pigment–protein complexes from the PSII light-harvesting complex [43,44,45]. Among the PSII partial complexes, OEC is more sensitive to heat [46]. In the present study, OEC inactivation was observed in the wild type after exposure to heat stress, as evidenced by the increase in *W*_K_ in the wild type. RC/CSo decreased in the wild type under heat stress, which meant that the amount of active PSII RCs greatly decreased. In addition, the decrease in *φ*_Eo_ and *φ*_Ro_ was more in the wild type than in the green mutant under heat stress, indicating higher inhibition of PSII electron transport. In contrast, in the green mutant, OEC was less affected by high temperature, and the lower decrease in active PSII RCs and lower inhibition of electron transport in response to high temperature could help the green mutant adapt to heat stress.

Heat dissipation is essential for protecting the leaf from stress-induced damage [28]. The NPQ process can harmlessly dissipate the excess energy, thus alleviating photodamage by reducing ROS production [47,48,49]. In the present study, the lower increase in NPQ in the wild type indicated lower heat dissipation capacity in response to heat stress, which may cause ROS accumulation. In contrast, the green mutant could be able to dissipate heat efficiently, reducing the risk of oxidative stress attacking the photosynthetic apparatus. ROS production, including single oxygen (^1^O_2_), superoxide (O_2_^−^), hydrogen peroxide (H_2_O_2_), and hydroxyl radical (OH^−^) production, is a sign of cellular injury caused by heat stress [3]. Superoxide, the product of a one-electron reduction in oxygen, is a ROS precursor and a mediator in oxidative chain reactions [50]. The superoxide content, measured as OFR, can reflect the ROS level in organisms. In this study, the OFR of the wild type increased much more than that of the green mutant under heat stress, indicating that it was subjected to severe oxidative stress. ROS can cause the autocatalytic peroxidation of membrane lipids, which is indicated by MDA, a product of the reaction [3]. After exposure to heat stress, MDA accumulated to a greater extent in the wild type than in the green mutant. The OFR and MDA of the green mutant showed a much lower increase under heat stress than the wild type, indicating its superior ability to maintain cell membrane integrity and minimize oxidative damage. To scavenge ROS, cellular antioxidant defenses that produce more antioxidant enzymes, such as SOD, POD, and CAT, are usually activated [6]. In this study, POD and CAT activities in the two strains increased after heat stress. The high POD and CAT activities in the green mutant suggest that it possessed an efficient antioxidant system for protection from oxidative stress and was able to inhibit ROS production in response to heat stress. The enhanced ROS detoxification capacity allowed the green mutant to better maintain its photosynthesis ability.

### 3.2. Low Temperature Stress in G. lemaneiformis

Chilling temperature can affect algal photosynthesis [51]. Low temperature inhibits carbon dioxide fixation, leading to ROS production, which in turn suppresses de novo D1 protein synthesis and reduces PSII repair, resulting in more severe damage to PSII [52]. In the present study, the OJIP fluorescence intensity of the two strains was lower under low temperature, suggesting the inhibition of photosynthetic performance. The greater decrease in *F*_V_/*F*_m_ and PI_abs_ values in the green mutant indicated that it had a greater degree of inhibition of photosynthetic performance.

However, the increase in OFR content was greater in the wild type than in the green mutant under low-temperature conditions, indicating that the green mutant has lower ROS levels than the wild type. Excessive ROS accumulation induces the production of aldehydes, which can cause genotoxic effects, such as lipid peroxidation [53]. The level of lipid peroxidation was much higher in the wild type than in the green mutant. The ROS and peroxidation levels of the green mutant remained low and increased after a low-temperature treatment, suggesting that the antioxidant potential of the green mutant may be higher under low-temperature conditions. The trend of increasing SOD activity was higher in the green mutant than in the wild type under low-temperature conditions. SOD is the first line of defense against ROS; the enzyme converts superoxide to hydrogen peroxide and oxygen [54]. The superoxide content and lipid peroxidation level were also lower in the green mutant than in the wild type under low temperatures. An increase in SOD activity was observed in *G. lemaneiformis* grown under low temperature, suggesting that reduced temperature enhances antioxidant defense [6]. There may be some mechanism in the green mutant to cope with the low-temperature stress, which deserves further investigation.

## 4. Materials and Methods

### 4.1. Sample Collection, Treatment, and Identification

The two *G. lemaneiformis* strains were obtained from cultivation rafts in Gaolv Aquaculture Co., Ltd., Rongcheng, Shandong, China (122.6° E, 37.2° N). The stable green-pigmented mutant was derived from the *G. lemaneiformis* cultivar 981 by spontaneous mutation and continuous selection for 3 years from 2019. After collection, the two *G. lemaneiformis* strains were first cultured in conical flasks with filtered seawater at 20 °C for 5 days at an irradiance of 72 μmol photons m^−2^ s^−1^ (12 h light:12 h dark).

ITS sequences were obtained for the green mutant and the wild type (through DNA extraction, PCR amplification, and sequencing) in addition to the seven sequences from GenBank. The evolutionary history was inferred by using the maximum likelihood method based on the general time reversible model [55]. The tree with the highest log likelihood (−6831.85) is shown. The percentage of trees in which the associated taxa clustered together is shown next to the branches. Initial tree(s) for the heuristic search were obtained automatically by applying Neighbor-Join and BioNJ algorithms to a matrix of pairwise distances estimated using the Maximum Composite Likelihood (MCL) approach and then selecting the topology with superior log likelihood value. A discrete Gamma distribution was used to model evolutionary rate differences among sites (five categories (+G, parameter = 1.9160)). The rate variation model allowed for some sites to be evolutionarily invariable ([+I], 16.84% sites). The tree is drawn to scale, with branch lengths measured in the number of substitutions per site. Codon positions included were 1st+2nd+3rd+Noncoding. There were a total of 1171 positions in the final dataset. Evolutionary analyses were conducted in MEGA7 [56].

### 4.2. Experimental Design

For temperature treatments, the two *G. lemaneiformis* strains were cultured at three temperatures—low (8 °C), medium (20 °C), and high (30 °C). The experimental temperature was selected according to the actual culture temperature of *G. lemaneiformis*. The culture was started when 3.0 g of fresh-weight algae was introduced into 18 conical flasks containing 1 L filtered seawater. Light conditions (intensity and period) for all treatments were the same as indicated above. Samples were cultivated for 7 days and harvested to assess physiological and biochemical responses under different temperature conditions. Replicate cultures (*n* = 3) were maintained at each treatment condition to avoid pseudoreplication.

### 4.3. Chlorophyll Fluorescence Measurements

Fast chlorophyll fluorescence intensity of the OJIP transient was measured under the FC1000-H fluorescence imaging system (Photon Systems Instruments Ltd., Brno, Czech Republic). All *G. lemaneiformis* samples were kept in the dark for 15–20 min before measurement to keep the PSII reaction centers fully open. The fluorescence intensity of the O, J, I, and P steps was recorded after 20 μs (F_O_), 2 ms (F_J_), 30 ms (F_I_), and 1000 ms (F_P_), respectively. The saturated light intensity of the OJIP transient was 3000 μmol m^−2^ s^−1^. Abbreviations, formulae, and definitions of the JIP-test parameters are listed in Table 1 [57,58,59].

### 4.4. Enzyme Activity Measurements

Oxygen free radical (OFR) and malondialdehyde (MDA) content, and SOD, CAT, and POD activities were measured using physiological assay kits (Suzhou Grace Biotechnology Co., Ltd., Suzhou, China) according to the manufacturers’ recommendations. All processes were biologically and temporally repeated in three independent and parallel experiments.

### 4.5. Statistics

All data are expressed as means ± standard deviation (SD, *n* = 3). Statistical analyses were performed using SPSS v.21.0 (IBM Corp., Armonk, NY, USA).The differences between treatments (HT and LT) and the control (MT) were considered significant if *p* < 0.05.

## 5. Conclusions

Compared with the wild type of *G. lemaneiformis*, the green mutant showed more tolerance to heat stress, as indicated by its higher photosynthetic performance and stronger antioxidant activity. In addition, the green mutant produced less ROS under low temperatures, which may have some potential to alleviate the damage caused by a decrease in photosynthetic performance. In conclusion, the green mutant has increased tolerance to high temperatures, laying the foundation for the selection of a resistant strain of *G. lemaneiformis*, while the green mutant may provide material for further genetic research and production applications as a superior algal strain.

## Figures and Tables

**Table 1:** Abbreviations, formulae, and definitions of JIP-test parameters.

Parameters	Definitions
*F*_O_ = *F*_20μs_	Minimum fluorescence, all PSII RCs are open
*F*_K_ = *F*_300μs_	Fluorescence intensity at the K-step (300 µs) of OJIP
*F*_J_ = *F*_2ms_	Fluorescence intensity at the J-step (2 ms) of OJIP
*F*_I_ = *F*_30ms_	Fluorescence intensity at the I-step (30 ms) of OJIP
*F*_m_ = *F*_P_	Maximum fluorescence at peak P of OJIP
*V*_J_ = (*F*_J_ − *F*_O_)/(*F*_m_ − *F*_O_)	Relative variable fluorescence at the J-step of the fluorescence induction curve
*V*_I_ = (*F*_I_ − *F*_O_)/(*F*_m_ − *F*_O_)	Relative variable fluorescence at the I-step of the fluorescence induction curve
*V*_K_ = (*F*_K_ − *F*_O_)/(*F*_m_ − *F*_O_)	Relative variable fluorescence at the K-step of the fluorescence induction curve
*W*_K_ = (*F*_K_ − *F*_O_)/(*F*_J_ − *F*_O_)	Damage to the oxygen-evolving complex
*M*o = 4(*F*_K_ − *F*_O_)/(*F*_m_ − *F*_O_)	Approximated initial slope (in ms^−1^) of the fluorescence transient
*F*_V_/*F*_m_	Maximum photochemical quantum yield
*φ*_Po_ = 1 − *F*_O_/*F*_m_ = *F*_V_/*F*_m_	Maximum quantum yield for primary photochemistry
*φ*_Eo_ = *F*_V_/*F*_m_ × (1− *V*_J_)	Quantum yield for electron transport
*φ*_Ro_ = *F*_V_/*F*_m_ × (1− *V*_I_)	Quantum yield of reduction of end electron acceptors of PSI
*Ψ*o = 1 − *V*_J_	Efficiency with which a trapped exaction can move an electron into the electron transport chain further than Q_A_
ABS/RC = (*M*o/*V*_J_)/(1/*φ*_Po_)	Light absorption flux (for PSII antenna chlorophyll) per RC
ETo/RC = (*M*o/*V*_J_) × (1 − *V*_J_)	Maximum electron transport flux (further than Q_A_^−^) per PSII RC
TRo/RC = (*M*o/*V*_J_)	Trapped (maximum) energy flux (leading to Q_A_ reduction) per RC
DIo/RC = ABS/RC − TRo/RC	Dissipation energy flux per PSII RC
RC/CSo = *φ*_Po_ × (*V*_J_/*M*o) × (ABS/CS)	Number of active RCs per CS
PI_abs_ = (RC/ABS) × [*φ*_Po_/(1 − *φ*_Po_)] × [*ψ*_o_/(1 − *ψ*_o_)]	Performance index on absorption basis

## Data Availability

The datasets used and/or analyzed during this study are available from the corresponding author upon reasonable request.

## Supplementary Materials

The following supporting information can be downloaded at: https://www.mdpi.com/article/10.3390/plants12081670/s1, Table S1: JIP-test parameters deduced from chlorophyll a fluorescence OJIP transient curves in the green mutant and wild type of *G. lemaneiformis* exposed to three temperature levels for 7 days.
