# Supplementary material for: Characterization of Chlorophyll Fluorescence and Antioxidant Defense Parameters of Two Gracilariopsis lemaneiformis Strains under Different Temperatures
Source: Plants (Basel). 2023 Apr 17;12(8):1670. doi: 10.3390/plants12081670 (PMC10146300; doi:10.3390/plants12081670)
Supplement: Supplementary file 1 [file plants-12-01670-s001.zip › plants-2275685-supplementary.pdf]

plants

Supplementary Materials

Table S1. Abbreviations, formulae, and definitions of JIP-test parameters. JIP-test parameters deduced from chlorophyll a fluorescence OJIP transient curves in the green mutant and wild type of G. lemaneiformis exposed to three temperature levels for 7 days.

	**Pigment mutant**			The wild type		
	**MT**	**HT**	**LT**	MT	HT	LT
W_K	0.504	0.467	0.430	0.417	0.478	0.541
F_V/F_m	0.330	0.355	0.258	0.284	0.269	0.243
φ_{Eo}	0.282	0.268	0.220	0.273	0.229	0.211
φ_{Ro}	0.232	0.248	0.193	0.218	0.197	0.196
ABS/RC	6.229	5.663	6.691	6.298	6.712	8.968
TRo/RC	2.017	1.866	1.719	2.124	2.178	2.163
ETo/RC	1.735	1.630	1.471	1.751	1.421	1.881
DIo/RC	4.212	2.897	4.972	4.174	5.417	6.805
RC/CSo	6.618	7.182	5.763	7.010	6.562	4.403
PI_{abs}	0.522	0.441	0.315	0.318	0.200	0.244

LT, low temperature 8°C; MT, medium temperature 20 °C; HT, high temperature 30 °C. MT was the control.
